# Anasarca Revealing Severe Cardiac Involvement Due to Behçet's Disease (BD): A Case Report

**DOI:** 10.7759/cureus.34532

**Published:** 2023-02-02

**Authors:** Jihane Smaali, Amal Charef, Mehdi Bamous, Jamal Fatihi, Taoufik Amezian

**Affiliations:** 1 Internal Medicine, Mohammed V Military Training Hospital, Rabat, MAR; 2 Faculty of Medicine and Pharmacy of Casablanca, Hassan II University, Casablanca, MAR; 3 Cardiac Surgery, Mohammed V Military Training Hospital, Rabat, MAR

**Keywords:** anasarca, tricuspid valve regurgitation, endomyocardial fibrosis, intracardiac thrombosis, behçet’s disease

## Abstract

Behçet's disease (BD) is a systemic vasculitis which is most often manifested by recurrent oral aphthosis, genital aphthosis, and ocular involvement with sometimes visceral damage, in particular neurological, digestive, vascular, or renal. We report the case of a 21-year-old man admitted for anasarca who revealed severe cardiac involvement associating endomyocardial fibrosis, intracardiac thrombi and involvement of the tricuspid valve in the context of BD diagnosed a posteriori. Cardiac involvement is exceptional during BD, especially as a mode of entry into the disease. It can be particularly severe, hence the need for early diagnosis, rapid and sometimes aggressive management. Close monitoring is also necessary in order to watch for the occurrence of visceral manifestations, particularly in young patients.

## Introduction

Behçet’s disease (BD) is a systemic vasculitis classified under the subgroup of variable vessel vasculitis [1]. The diagnosis is based on clinical features such as recurrent oral aphtosis, genital ulcers, and uveitis [2]. Cardiac involvement during BD is rare; it would concern more young male subjects between 20 and 40 years of age [3] and all tunics can be affected such as pericarditis, myocarditis, and valvular involvement [4]. We report the observation of a young patient who presented with anasarca and in whom the diagnosis of BD with severe cardiac involvement was made and raised through this clinical case the diagnostic and therapeutic difficulties of this exceptional type of damage.

## Case presentation

A 21-year-old man with a history of bronchial asthma and recurrent oral aphtosis was admitted for exploration of a state of anasarca. For two months, he had exhibited symptoms of NYHA stage II heart failure: dyspnea induced by usual physical activities and a progressive increase in the volume of the lower limbs and abdomen evolving in a context of apyrexia and asthenia. On clinical examination, there were signs of right heart failure, ascites of great abundance, lower limb edema, and decreased breath sounds on the right side due to right pleural effusion of moderate abundance as well as genital and cutaneous ulcers.

 On biological assessment, there was a discreet inflammatory syndrome with sedimentation rate 35 mm/h and C-reactive protein 29 mg/L, microcytic anemia (hemoglobin 10.7 g/dL), hepatic cytolysis and cholestasis (aspartate aminotransferase: 53 UI, alanine aminotransferase: 71 UI, alkaline phosphatase: 103 UI, gamma-glutamyl-transferase: 98 UI), prothrombin: 65%, albumin: 32 g/L, renal function was normal and his 24-h urine protein test was negative (Table 1).

**Table 1:** Laboratory results. ESR, erythrocyte sedimentation rate; CRP, C-reactive protein; ASAT, aspartate aminotransferase; ALAT, alanine aminotransferase; ALP, alkaline phosphatase; GGT, gamma glutamyl transferase; TP, prothrombin; IU/l, international units/l

Biological parameters	Results	Reference values	Units of measurement
ESR	35	<15	mm/h
CRP	29	<5	mg/L
Hemoglobin	10,7	13-17	g/dL
ASAT	53	<35	IU/L
ALAT	71	<40	IU/L
ALP	130	32-91	IU/L
GGT	98	<50	IU/L
TP	65	70-100	%
Albumin	32	35-50	g/L

 Bacteriological samples (aero-anerobic blood cultures and fungicultures, cytobacteriological urine exam, coproculture), Koch’s Bacillus sputum, quantiferon, and serologies (human immunodeficiency virus, cytomégalovirus, and hepatitis B and C) were negative as well as the immunological assessment (antinuclear antibodies, anti-neutrophil cytoplasm antibodies).

**Figure 1:**
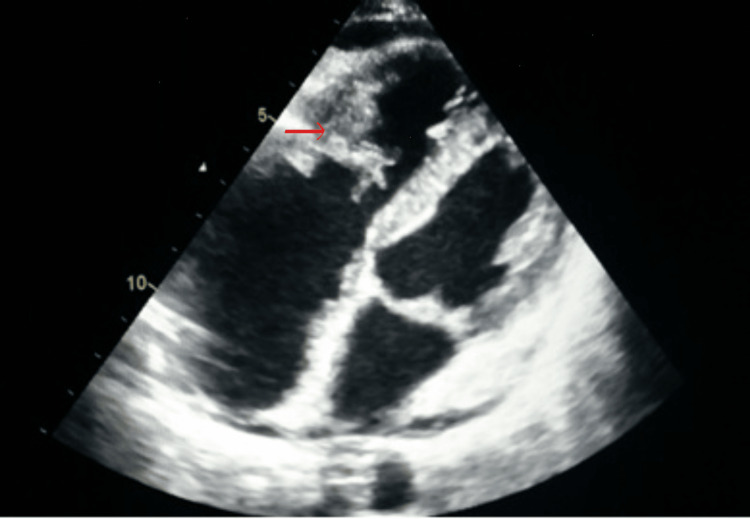
Four-cavity echocardiographic image showing nodular formations infiltrating the tricuspid valve and the RA. RA, right atrium

The transthoracic echocardiogram showed a severe pericardial effusion, masses lining the right ventricle (RV) which was slightly dilated and the roof of the right atrium (RA), a thickened tricuspid valve with severe regurgitation (Figure 1), the left ventricle ejection fraction was preserved and the other heart valves were unharmed. A complement by transesophageal echocardiogram made it possible to confirm the nodular lesions lining the RV and the RA suggestive of either thrombi or tumors as well as defects of the tricuspid valve whose septal leaflet appeared thinned with chordae rupture.

**Figure 2:**
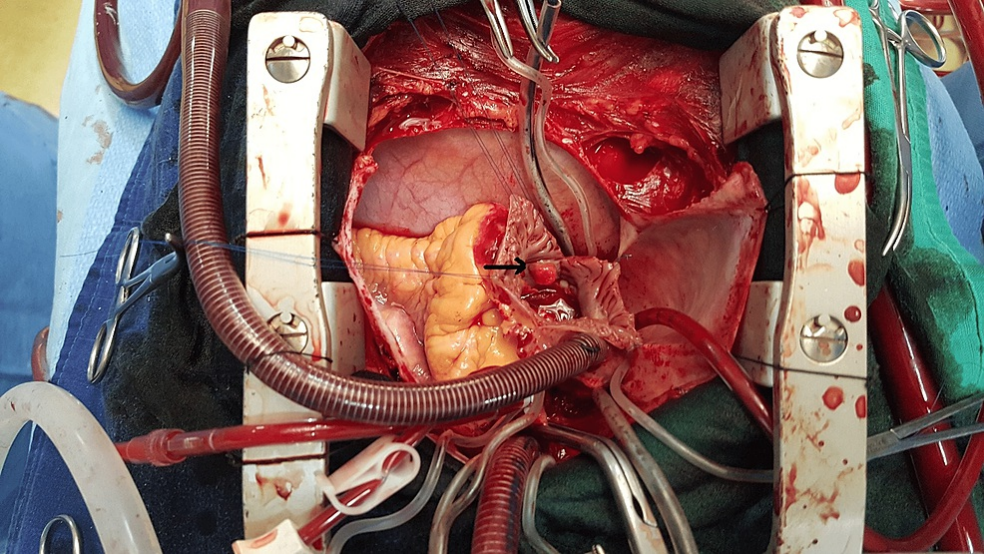
Intraoperative image: right atriotomy revealing a hanging mass at the interatrial septum.

Pericardocentesis brought back 700 cc of sterile transudative serohematic fluid. Faced with this aspect of cardiac masses and infiltration of the tricuspid valve, additional cardiac magnetic resonance imaging was carried out to specify the tissue or thrombotic nature of the masses and which objectified thickened tricuspid valve and isointense masses in the roof of RA and RV, the coronal T1 post-gadolinium demonstrated as isointense with late enhancement with gadolinium. Surgery was indicated after clinical stabilization. The right atriotomy made it possible to visualize a polypoid tumor formation attached to the lower edge of the interatrial septum, 1 cm in diameter (Figure 2) with a whitish coating lining the RA and RV and a thickened tricuspid valve with thinned septal leaflet. Tricuspid valve replacement by mechanical prothesis n°29, Sorin ,was performed due to severe tricuspid valve regurgitation and right ventricle dysfunction with resection of the RA mass.

The anatomopathological study of the mass revealed a fibrous connective tissue with a grossly nodular appearance, the site of diffuse fibrinoid necrosis with blood clot. Diagnosis of reorganized thrombus at the level of the RA associated with endomyocardial fibrosis and damage to the tricuspid valve in the context of BD was made due to the association of recurrent oral aphtosis (more than three episodes per year), genital aphtosis, and the thrombotic nature of the mass taken from the RA as well as the inflammatory and sterile nature of the biopsy samples from the tricuspid valve that was replaced.

## Discussion

Our patient presented a state of anasarca on laminar tricuspid insufficiency and thrombi of the right cavities as well as endomyocardial fibrosis realizing severe and exceptional cardiac involvement during BD. The diagnosis of BD was made according to the International Criteria for BD [2], the most used score in clinical practice.

Cardiac involvement during BD is rare, its prevalence would be approximately 1%-6% in clinical series [3], and 16.5% in an autopsy series [4] -- it would concern more young male subjects originating from around the Mediterranean [4]. All tunics can be affected (pericarditis, myocarditis, ventricular aneurysms, endomyocardial fibrosis, myocardial infarction, etc.) with a high frequency of benign acute pericarditis contemporaneous with an inflammatory flare-up [5-6]. Geri et al. reported a higher frequency of cardiac involvement in subjects with BD compared to healthy controls, it concerns 6% of patients with pericarditis in 38% of cases, endocarditis in 26%, 19% intracardiac thrombus, 17% myocardial infarction ,7% endomyocardial fibrosis, and 2% myocardial aneurysm [5].

The diagnosis of intracardiac thrombosis is often difficult because it can simulate a tumoral lesion, in particular an angiosarcoma, especially if contrast uptake on MRI as in our patient, and especially when they precede the diagnosis of BD, which is the case in 50% of cases approximately [7].

In the study of Teheran University of Medical Sciences in which 7650 patients were diagnosed with BD were analyzed, 47 patients manifested cardiac involvement: myocardial infarction in 23.4%, pericarditis in 21.3%, heart failure in 12.8%, one patient (2.1%) developed intracardiac thrombosis and valvular involvement was noted in 6.1% [6].

Endomyocardial fibrosis is an exceptional condition that can be discovered incidentally on echocardiography and is sometimes associated with intracardiac thrombosis or valvulopathy. Valvular involvement can pose a problem of differential diagnosis with rheumatic valvulopathy, especially since the two pathologies have common epidemiological characteristics [8].

The combination of these three disorders is exceptional and particularly severe. The treatment of this type of damage remains empirical and is based on expert opinions with a low level of evidence. It calls for combined treatments with corticosteroids, immunosuppressants, biological drugs [3], and sometimes anticoagulants and, more rarely, surgery, which would be fraught with complications and high mortality [7, 9].

In our patient, the initiation of a treatment combining diuretics, corticosteroids, and anticoagulants allowed clinical stabilization but given the importance of the defects of the tricuspid valve, a valve replacement was necessary but the outcome was fatal following postoperative infectious complications.

## Conclusions

This case illustrates the diagnostic and therapeutic difficulties of cardiac involvement in BD which would concern more young subjects and which sometimes constitutes the mode of revelation of the disease. Advances in terms of imaging with the contribution of MRI and the increasingly widespread use of TNF alpha inhibitors during severe manifestations of BD allow us to glimpse an improvement in the prognosis of these attacks.
